# The Importance of Multisystem Evaluation in Diagnosing Birt–Hogg–Dubé Syndrome: A Case Report

**DOI:** 10.5152/eurasianjmed.2024.24499

**Published:** 2024-10-01

**Authors:** Alperen Aksakal, Zeynep Karaca Ural, Latifullah Jalal, Gizem Çil, Buğra Kerget, Ömer Araz, Elif Yılmazel Uçar, Leyla Sağlam

**Affiliations:** 1 Department of Pulmonary Medicine, Atatürk University School of Medicine, Erzurum, Türkiye; 2 Department of Dermatology, Atatürk University School of Medicine, Erzurum, Türkiye

To the Editor,

Birt–Hogg–Dubé syndrome (BHDS) is a rare genetic condition caused by mutations in the folliculin (FLCN) gene. It is often characterized by skin lesions, lung cysts, and the development of both benign and malignant tumors in various organs.^1^ The wide clinical spectrum of this syndrome poses challenges in its diagnosis and management.

A 70-year-old male patient with a known diagnosis of chronic obstructive pulmonary disease and a history of pulmonary thromboembolism and deep vein thrombosis was admitted to the emergency room with complaints of shortness of breath, coughing, and inability to produce sputum. Physical examination revealed multiple 1-4 mm, dome-shaped, skin-colored bumps on the face, neck, postauricular region, and anterior chest wall (Figure 1). Decreased bilateral breath sounds were noted on auscultation, both anteriorly and posteriorly. Written informed consent was obtained from the patient who agreed to take part in the study. No other pathologies were observed in the physical examination. Increased levels of C-reactive protein, hemoglobin, and hematocrit were noted in the blood parameters. Other parameters were normal. A posterior–anterior chest x-ray showed a bilateral bullous appearance. Thorax computed tomography revealed numerous cystic lesions, with the largest being approximately 70 mm in size, in the lateral segment of the lower lobe of the right lung. These cysts had round, oval, and lentiform morphologies, predominantly located in the right basal segments, and were thin-walled, unilocular, multilocular, and septate, situated in subpleural and peribronchovascular areas (Figure 2). Histopathology of a papule excised from the anterior chest wall confirmed fibrofolliculoma. The patient was not tested for mutations in the FLCN gene.

Birt–Hogg–Dubé syndrome is characterized by benign skin lesions predominantly localized on the face and neck, pulmonary cysts, and an elevated risk of renal malignancy. Birt–Hogg–Dubé syndrome presents a triad of dermatological, pulmonary, and renal disease components.^2^ This complex syndrome includes a variety of lung cysts of various sizes and their potential complications, such as spontaneous pneumothorax, alongside renal tumors of different histological types and typical skin lesions, notably fibrofolliculomas, trichodiscomas, and acrochordons.^3^

The European Birt–Hogg–Dubé Consortium has outlined diagnostic criteria for BHDS, divided into major and minor categories. Major criteria are ≥5 fibrofolliculoma or trichodiscoma lesions occurring in adulthood, with at least one having a histopathological diagnosis, or a mutation in the FLCN gene. Minor criteria encompass multiple lung cysts, renal malignancy, and a first-degree relative with BHDS (Table 1). For a BHDS diagnosis, patients must meet either one major criterion or 2 minor criteria, underscoring the need for a multidisciplinary diagnostic approach due to the syndrome’s varied clinical presentation.^4^ This approach was pivotal in our patient's case, where dermatological consultations and pathology results played significant roles in assessing skin lesions for diagnosis. Additionally, the radiological examination of lung findings guided the diagnostic process.

In our patient's case, as a result of a detailed evaluation of the diagnostic findings, a major and a minor criterion of critical importance for BHDS were identified. Due to the syndrome's genetic nature, screening family members of diagnosed patients is recommended to facilitate early diagnosis and management of potentially affected individuals.^5^

In conclusion, BHDS represents a genetic, dermatological, pulmonary, and renal multifaceted disease. Its diagnosis and management necessitate a multidisciplinary approach, where early detection significantly influences patient outcomes. This case report aims to enhance awareness of BHDS's diagnostic and management challenges and underscores the pivotal role of a multidisciplinary approach.

## Data Availability Statement

The data that support the findings of this study are available on request from the corresponding author.

## Figures and Tables

**Figure 1.**
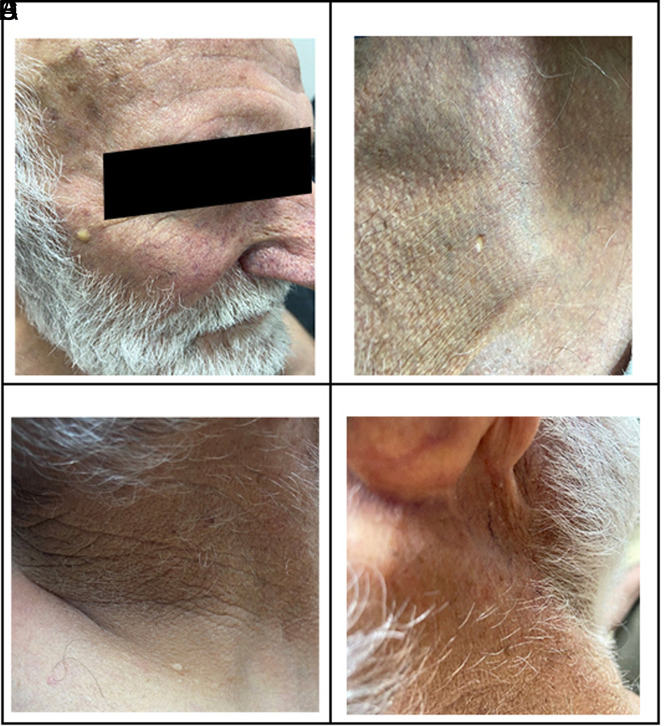
Skin-colored papular lesions on (A) face, (B) anterolateral neck, (C) posterolateral neck, and (D) postauricular region.

**Figure 2.**
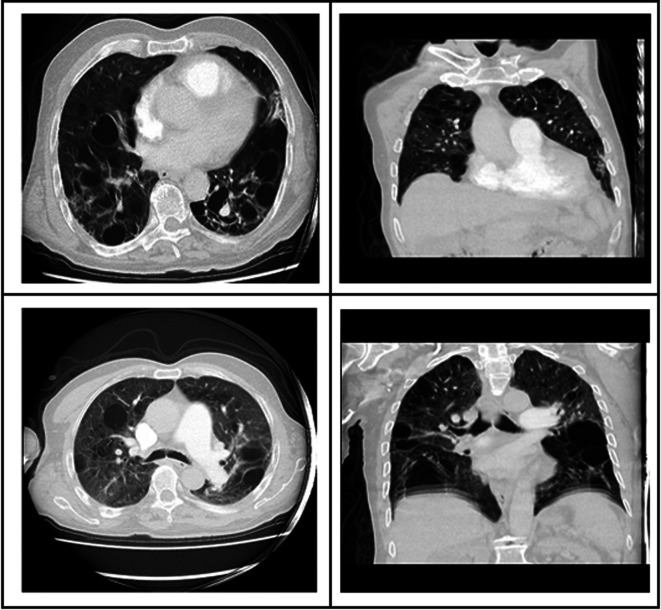
Widespread centriacinar emphysematous hyperinflation in each lung and thin-walled air cysts, approximately 6 cm in size, parenchymal distortions, and traction bronchiectasis expansions in the lower lobe of the elderly right lung.

**Table 1.** European Birt–Hogg–Dubé Syndrome Diagnostic Criteria

Major Criteria	Minor Criteria
≥5 fibrofolliculomas or trichodiscomas, at least one of which was diagnosed histopathologically in adulthood	Multiple lung cysts (bilaterally and basally located ± spontaneous pneumothorax)
Mutation in the FLCN gene	Kidney cancer (before age 50 or multifocal, bilateral or mixed chromophobe, oncocytic histopathology)
	Birt–Hogg–Dubé syndrome in first-degree relatives
To be diagnosed with Birt–Hogg–Dubé syndrome, patients must meet 1 major or 2 minor criteria	
